# Evaluation of productivity losses due to premature mortality from colorectal cancer

**DOI:** 10.1371/journal.pone.0244375

**Published:** 2020-12-23

**Authors:** Josep Darbà, Alicia Marsà

**Affiliations:** 1 Department of Economics, Universitat de Barcelona, Barcelona, Spain; 2 Department of Health Economics, BCN Health Economics & Outcomes Research S.L., Barcelona, Spain; Charite Universitatsmedizin Berlin, GERMANY

## Abstract

Cancer is responsible annually for around 27% of all deaths in Spain, 15% of which are caused by colorectal cancer. This malignancy has increased its incidence considerably over the past years, which surely impacts global productivity losses. The evaluation of lost productivity due to premature mortality provides valuable information that guides healthcare policies into the establishment of prevention and screening programs. The purpose of this study was to assess the productivity losses from premature deaths due to colorectal cancer over a ten year period (2008–2017). The costs derived from premature mortality due to this highly prevalent cancer were estimated using data on mortality, age- and sex-specific reference salaries and unemployment rates in Spain via the human capital approach. Between 2008 and 2017, 15,103 persons died per year from colorectal cancer, representing almost 15% of all cancer-related deaths. Annually, 25,333 years of potential productive life were estimated to be lost on average, 14,992 in males and 10,341 in females. Productivity losses summed €510.8 million in in 2017, and the cancers of the colon and rectum accounted for 9.6% of cancer-related productivity losses in 2017 in Spain. Colorectal cancer has an important weight in terms of productivity losses within the Spanish population, consequently, prevention and early detection programmes should be promoted and implemented to achieve significant reductions in mortality and productivity losses.

## Introduction

Cancer is the second most common cause of death in Europe [1], with 1.93 million people estimated to die from this disease in 2018 [2]. In Spain, cancers rank as the second leading cause of mortality, being responsible annually for around 27% of all deaths [3], 15% of which were caused by colorectal cancer. Additionally, while the incidence of certain cancers, as lung cancer in Spain is decreasing, the incidence of colorectal cancer has maintained an increasing tendency as measured between the years 1993 and 2015, reaching an age-standardised incidence rate (Europe) of 77.8 in men and 42.0 in women per 100,000 [4]. Colorectal cancer mortality in Spain increased between 1985 and 2004, with higher increases in males versus females and in older age groups versus younger ones [5]. Hence, it is of interest to analyse the economic burden that this cancer type represents for the Spanish society.

To estimate the economic burden of cancer, it is essential to conduct economic evaluations that will guide healthcare policies and prevention and screening programs. These evaluations include direct costs (medical and household expenditures, informal care…) and indirect costs (reduced or lost productivity due to morbidity or mortality) of disease [6]. In terms of indirect costs, one of the most recognised methods for the estimation of the social and economic losses attributed to a disease is the measurement of disability-adjusted live years (DALYs), where one DALY is equivalent to one year of healthy life lost [7]. Equally, the years of productive life lost (YPPLL) can be calculated; which allows an assessment of the productivity losses due to premature mortality [8]. In the calculation of YPPLL, it is considered that years from death to projected retirement are lost due to the disease.

In Europe, 50,168,779 DALYs were attributed to cancer in 2016, 11.15% of those to colorectal cancer, and 48,784,557 years of life were lost [7]. Earlier calculations estimated 302,089 YPPLL due to cancer in Spain, with losses around €2.5 billion [9]. The importance of colorectal cancer in Spain has been widely described, given its prevalence within the Spanish population; it is also the second cancer responsible for the most number of deaths after lung cancer [3]. Additionally, a multicentre cross-sectional study in Spain the year 2014 showed that 50% of the population affected by this cancer were between 65 and 79 years of age, while 30% were younger than 65 years [10], which has a direct impact on productivity loss calculations.

This study aims to assess the productivity losses derived from premature deaths due to colorectal cancer in Spain within a ten year period.

## Materials and methods

Several methods are available for the calculation of the indirect costs of disease. Herein, the human capital approach was used to measure productivity losses due to colorectal cancer in Spain. This method estimates the indirect costs that the disease represents for the individual, family, society or employer via the calculation of the income and productivity of an individual that are prevented when premature death occurs [11].

Statistics on mortality and salaries were extracted from the Spanish National Statistics Institute (INE) [12, 13]. The years of potential productive life lost (YPPLL) due to premature mortality from colorectal cancer were estimated by multiplying the number of colorectal cancer-specific deaths for a given age group by the expected productive years remaining for each group. Retirement age was fixed at 65 years, the legal age of retirement in Spain. The costs of premature mortality were estimated using age- and sex-specific annual wages from death age to age of retirement were used. YPPLL was corrected per age- and gender-specific unemployment rates and an annual discount rate of 3% was applied to future income values in the baseline analysis [14]. A sensitivity analysis was conducted considering two alternative discount rates (0% and 6%) following recommendations for Spain [15, 16].

The same calculations were conducted considering persons dead per all cancers as registered in the Spanish National Statistics Institute, which includes cancers of the lung, breast, pancreas, prostate, stomach, liver, bladder, kidney, haematological cancers, cancers of the nervous system, oesophagus, melanoma, cancers of the reproductive system, oral cavity, larynx, oropharynx, nasopharynx, gallbladder, mesothelioma, thyroid and Kaposi sarcoma. Ethics committee approval and consent were not required for this study.

## Results

Between 2008 and 2017, 15,103 persons died per year from colorectal cancer, summing a total of 151,032 persons (Table 1). A 3:2 male/female ratio was measured constantly over the years. Cancer of the colon and rectum represented on average 14.91% of all cancer-related deaths, and a mean of 18.27% of those deaths were at working age, being this parameter slightly higher in males. Large fluctuations in the number of deceased individuals have been registered over the years, yet, overall, a more pronounced increase is measured the first half of the study period.

**Table 1:** Indicators of deaths, deaths at working age and years of potential productive life lost (YPPLL) due to colorectal cancer.

Year	2008	2009	2010	2011	2012	2013	2014	2015	2016	2017
Number of deaths										
*males*	7,987	8,365	8,816	8,994	9,362	9,235	9,260	8,818	9,430	9,103
*females*	5,829	5,873	6,067	6,388	6,242	6,364	6,216	6,013	6,363	6,307
% of deaths at working age (16–65)										
*males*	20.23	20.43	19.29	19.62	20.12	19.61	19.56	16.58	18.42	18.57
*females*	17.98	17.18	17.57	17.44	17.83	17.54	16.68	13.35	16.74	16.20
% of colorectal cancer deaths vs. all cancers										
*males*	13.24	12.88	14.25	17.29	8.58	14.51	18.51	13.02	13.75	15.26
*females*	14.68	15.59	14.76	18.81	1.94	15.11	15.67	13.77	15.23	14.09
YPPLL total	24,732	25,649	25,576	26,457	27,706	26,701	26,484	20,585	25,316	24,126
*males*	14,103	15,137	14,673	15,535	16,772	15,618	15,908	12,801	14,926	14,450
*females*	10,629	10,512	10,903	10,922	10,934	11,083	10,576	7,784	10,390	9,676

In terms of lost productivity, 25,333 YPPLL annually were estimated during the study period; 24,126 on 2017.

**Fig 1:**
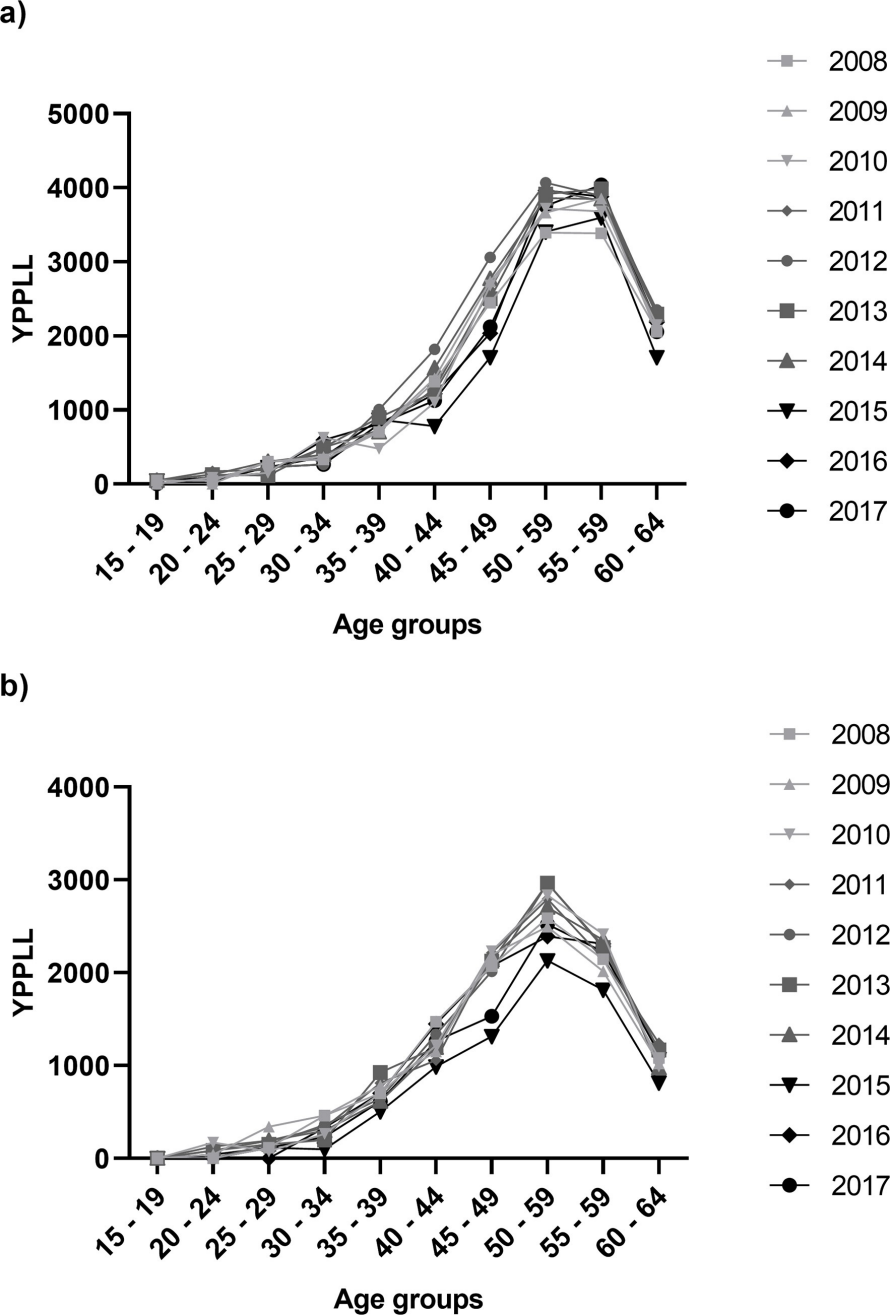
Years of potential productive life lost (YPPLL) due to premature mortality from colorectal cancer per age groups between 2008 and 2017 in males (a) and females (b).

The age distribution of YPPLL per year were analysed for males and females. Premature mortality due to colorectal cancer accounted for the highest values of YPPLL in the age period between 50 and 59 years in both males and females (Fig 1), which impacts directly productivity costs. Contrarily, fewer YPPLL are measured in younger individuals, this can be attributed to the lower mortality of the disease in this age group versus that in older individuals.

The calculation of losses due to premature mortality was accomplished via the projection of productivity losses per year to retirement age, adjusting all calculations for age- and sex-specific annual wages. Three estimations were obtained, a baseline, and two resulting from the sensitivity analysis. Over the ten-year study period, the estimated losses due to colorectal cancer were €5.2 billion, €510.8 million when only considering the year 2017 (Table 2). The sensitivity analysis determined a range of total costs between €5.1 and €5.3 billion.

**Table 2:** Productivity losses (in millions) due to colorectal cancer (a 3% discount rate was used in the baseline analysis; 0% and 6% discount rates were used in the sensitivity models).

Year	Productivity losses (baseline 3%)	Productivity losses (0%)	Productivity losses (6%)
2008	555.6	560.8	541.6
2009	548.5	553.5	534.7
2010	531.9	536.5	518.5
2011	540.3	545.0	526.7
2012	550.5	555.6	536.7
2013	515.5	519.8	502.6
2014	516.5	521.1	503.5
2015	416.1	419.2	405.7
2016	520.1	524.4	507.0
2017	510.8	514.6	498.0
Total	5,205.8	5,250.4	5,075.1

The percentage of the costs of lost productivity due to colorectal cancer in relation to all cancers was also calculated. The cancers of the colon and rectum accounted for 9.6% of cancer-related productivity losses in 2017 (Table 3). No clear tendencies were observed over time.

**Table 3:** Percentage of productivity losses (in millions) due to colorectal cancer vs. all cancers (a 3% discount rate was used in the baseline analysis; 0% and 6% discount rates were used in the sensitivity models).

Year	% productivity losses (baseline 3%)	% productivity losses (0%)	% productivity losses (6%)
2008	10.1	9.9	10.1
2009	9.4	9.3	9.4
2010	12.5	12.3	12.5
2011	9.5	9.3	9.5
2012	13.0	12.8	13.0
2013	12.7	12.4	12.7
2014	10.1	9.9	10.1
2015	8.0	7.9	8.0
2016	10.6	10.5	10.6
2017	9.6	9.5	9.6

## Discussion

The estimation of productivity loss provides a general vision of disease burden that is crucial for resource allocation and establishment of cancer prevention and detection programs [17]. Worldwide, cancer represents one of the major burdens to confront and colorectal cancer contributes increasing this sum [9]. Colorectal cancer is the second leading cancer-related mortality cause worldwide and also in Spain [3]. In 2017, 9,103 males and 6,307 females died from this cancer type in this country, with a percentage of deaths at working age that decreased over the study period; 17.6% of deaths occurred during working age, which impacts labour productivity.

Estimations of productivity losses due to premature cancer mortality worldwide suggest annual losses up to hundreds of billions of dollars, with 196.3 million DALYs calculated in 2013 [18]. One study in the United States projected cancer productivity losses that would increase to $148 billion in 2020 [19]; in the European Union, cancer was estimated to be annually responsible for productivity losses between €42.6 and €75.5 billion depending on the model, with 8–10% of those losses related to colorectal cancer [1, 20].

It may be difficult to compare studies due to methodological variations that have been previously described [21, 22]; nonetheless, this study results appear comparable to previous Spanish and global cancer burden evaluations. Peña-Longobardo et al. measured €2.5 billion in productivity losses due to cancer in Spain in 2009 [23]; in a direct comparison, our study suggests that up to 22% of those losses could be due to colorectal cancer. A similar study estimated €2.1 billion in mortality productivity losses for women with breast cancer in Spain in 2014 and 27,633 YPPLL the same year [16], a seemingly elevated figure, partly justified by the prevalence of breast cancer among women. Similarly, lung cancer was responsible for 36,246 YPPLL in males and 23,035 YPPLL in females in 2017, and 899 and 284 million euros in losses in males and females, respectively, in 2017 [24].

The interest of such studies is justified with the formulation of specific measures that aim to prevent, detect and reduce cancer incidence, and thus its costs. The implementation of colorectal cancer screenings in Spain started in 2005 two years after the European Colorectal Cancer Screening recommendation to perform pilot studies was issued [25]. The year 2014 such programmes were included in reimbursement lists and covered by the National healthcare system [26], which has allowed to increase colorectal cancer detection rates. Such screenings are aimed at the population aged 50 to 69 years, and even though only 43.92% of target individuals participated in the screening programmes in 2011, overall participation rates follow an increasing tendency over time [27]. Cancer detection rate was 2.3% in 2012 and 72.2% of cancers were detected in early stages, which validates the utility of screenings allowing early detection and subsequent treatment [27]. Still, the prevalence of this cancer and the productivity losses derived make evident the need to increase participation rates, especially among males, also considering the long-term costs that are derived from its treatment [28]. Direct medical costs linked to colorectal cancer reach figures around the €41,550 per patient in long-term costs for patients in stage III [28]. In addition, estimates point out that an annual 1% reduction in cancer mortality would decrease productivity losses by €8.5 billion over 20 years [29].

Equally, the promotion of healthier behaviours is recommended aiming to decrease colorectal cancer incidence, as regular physical activity, the consumption of fibre, fruits and vegetables, folate, calcium, vitamin D, vitamin B6 and magnesium [30].

It is plausible that a number of limitations may have influenced the results of this study. The lack of data on disease incidence in both males and females over the study period has limited data interpretation. The human capital approach is the majority method used for the calculation of indirect costs of disease via productivity losses, although it presents a series of restrictions. The assumption that future earnings translate into productivity has been questioned, together with the consideration that productivity lost is not covered by another individual [6].

## Conclusions

Altogether, the productivity costs due to colorectal cancer mortality have been evaluated and updated. Deaths from colorectal cancer represented almost 15% of all cancer-related deaths, with 25,333 YPPLL estimated annually and accounting for €510.8 million in productivity losses in 2017. This data underlines the burden of colorectal cancer on the Spanish population, providing novel data on death number, trends and productivity losses that this cancer type represents. This may assist decision makers in the allocation of resources, prioritising early detection screening programmes that possibly yield substantial reductions in mortality and productivity loss.

## List of abbreviations

DALYs: Disability-adjusted life-years
INE: Spanish national statistics institute (Instituto Nacional de Estadística)
YPPLL: Years of potential productive life lost
